# Hydrogen-Oxidizing Bacteria Are Abundant in Desert Soils and Strongly Stimulated by Hydration

**DOI:** 10.1128/mSystems.01131-20

**Published:** 2020-11-17

**Authors:** Karen Jordaan, Rachael Lappan, Xiyang Dong, Ian J. Aitkenhead, Sean K. Bay, Eleonora Chiri, Nimrod Wieler, Laura K. Meredith, Don A. Cowan, Steven L. Chown, Chris Greening

**Affiliations:** a School of Biological Sciences, Monash University, Clayton, VIC, Australia; b Centre for Microbial Ecology and Genomics, Department of Biochemistry, Genetics and Microbiology, University of Pretoria, Pretoria, South Africa; c Department of Microbiology, Biomedicine Discovery Institute, Monash University, Clayton, VIC, Australia; d School of Marine Sciences, Sun Yat-Sen University, Zhuhai, China; e Geological Survey of Israel, Jerusalem, Israel; f School of Natural Resources and the Environment, University of Arizona, Tucson, Arizona, USA; University of California San Diego

**Keywords:** carbon fixation, desert, hydrogen, hydrogenase, primary production, trace gas

## Abstract

How the diverse bacterial communities inhabiting desert soils maintain energy and carbon needs is much debated. Traditionally, most bacteria are thought to persist by using organic carbon synthesized by photoautotrophs following transient hydration events. Recent studies focused on Antarctic desert soils have revealed, however, that some bacteria use atmospheric trace gases, such as hydrogen (H_2_), to conserve energy and fix carbon independently of photosynthesis. In this study, we investigated whether atmospheric H_2_ oxidation occurs in four nonpolar desert soils and compared this process to photosynthesis. To do so, we first profiled the distribution, expression, and activities of hydrogenases and photosystems in surface soils collected from the South Australian desert over a simulated hydration-desiccation cycle. Hydrogenase-encoding sequences were abundant in the metagenomes and metatranscriptomes and were detected in actinobacterial, acidobacterial, and cyanobacterial metagenome-assembled genomes. Native dry soil samples mediated H_2_ oxidation, but rates increased 950-fold following wetting. Oxygenic and anoxygenic phototrophs were also detected in the community but at lower abundances. Hydration significantly stimulated rates of photosynthetic carbon fixation and, to a lesser extent, dark carbon assimilation. Hydrogenase genes were also widespread in samples from three other climatically distinct deserts, the Namib, Gobi, and Mojave, and atmospheric H_2_ oxidation was also greatly stimulated by hydration at these sites. Together, these findings highlight that H_2_ is an important, hitherto-overlooked energy source supporting bacterial communities in desert soils. Contrary to our previous hypotheses, however, H_2_ oxidation occurs simultaneously rather than alternately with photosynthesis in such ecosystems and may even be mediated by some photoautotrophs.

**IMPORTANCE** Desert ecosystems, spanning a third of the earth’s surface, harbor remarkably diverse microbial life despite having a low potential for photosynthesis. In this work, we reveal that atmospheric hydrogen serves as a major previously overlooked energy source for a large proportion of desert bacteria. We show that both chemoheterotrophic and photoautotrophic bacteria have the potential to oxidize hydrogen across deserts sampled across four continents. Whereas hydrogen oxidation was slow in native dry deserts, it increased by three orders of magnitude together with photosynthesis following hydration. This study revealed that continual harvesting of atmospheric energy sources may be a major way that desert communities adapt to long periods of water and energy deprivation, with significant ecological and biogeochemical ramifications.

**Author Video**: An author video summary of this article is available.

## Abstract (precis)

Desert ecosystems, spanning a third of the earth’s surface, harbor remarkably diverse microbial life despite having a low potential for photosynthesis. In this work, we reveal that atmospheric hydrogen serves as a major previously overlooked energy source for a large proportion of desert bacteria. We show that both chemoheterotrophic and photoautotrophic bacteria have the potential to oxidize hydrogen across deserts sampled across four continents. Whereas hydrogen oxidation was slow in native dry deserts, it increased by three orders of magnitude together with photosynthesis following hydration. This study revealed that continual harvesting of atmospheric energy sources may be a major way that desert communities adapt to long periods of water and energy deprivation, with significant ecological and biogeochemical ramifications.

## INTRODUCTION

Spanning a third of land surfaces, deserts are extensive terrestrial biomes that are expanding due to multiple anthropogenic pressures (1, 2). Organisms residing in these ecosystems endure severe and prolonged drought interspersed with infrequent hydration pulses, often in combination with other physicochemical pressures (3–6). Drought limits the abundance and productivity of oxygenic photoautotrophs, namely, cyanobacteria, microalgae, and plants, which require water as an electron donor. Low supplies of organic carbon, combined with impaired substrate diffusion and membrane transport, in turn limit the abundance of chemoheterotrophic microorganisms and fauna (6, 7). The extents of nutrient limitation vary: semiarid deserts experience considerable precipitation and subsequent productivity, albeit counterbalanced by evapotranspiration, whereas hyperarid deserts can be deprived of rainfall over decadal scales (8, 9). In spite of these pressures, cultivation-independent surveys have shown that microorganisms are moderately abundant in most desert soils and can be as diverse as those of humid soils (10, 11). Cyanobacteria and microalgae are dominant in certain niches within desert ecosystems, such as biocrusts and hypoliths (12–14). In contrast, these primary producers are low in abundance in exposed surface soils, which are instead dominated by chemoheterotrophs from phyla such as *Actinobacteriota*, *Acidobacteriota*, and *Proteobacteria* (10, 15, 16). Most of these bacteria are thought to live in dormant states during dry periods (6, 7, 17); while they have detectable transcriptional and metabolic activity (18–20), cells primarily expend energy for maintenance rather than growth. Rainfall pulses stimulate microbial activity and succession over short timescales, and the frequency and intensity of these events are thought to be major factors determining the composition and function of desert microbial communities over longer timescales (21–28).

One area of debate is what energy sources sustain the chemoheterotrophic majority in desert surface soils. As we recently reviewed, two distinct but not necessarily competing hypotheses have been developed (6). The “energy reserve hypothesis” proposes that these communities are sustained by the organic carbon that becomes transiently available following hydration events, through photoautotrophic activity and other processes. Chemoheterotrophs are hypothesized to store some of the organic carbon released following hydration events and use it as an energy source to maintain themselves in predominantly dormant states during subsequent long-term desiccation (6, 29–31). Hence, they are adapted to a feast-and-famine lifestyle. More recently, the “continual energy-harvesting hypothesis” proposed that microorganisms can survive independently of photoautotrophs. They do so by harvesting two alternative exogenous energy sources that are ubiquitously available at the soil interface: sunlight and atmospheric trace gases. Some proteobacterial lineages present in deserts have the capacity to transduce solar energy into a proton motive force using energy-converting rhodopsins (32, 33) or anoxygenic photosynthesis (34–36). Likewise, multiple actinobacterial lineages that predominate in deserts are proposed to express high-affinity enzymes to use atmospheric trace gases as alternative electron donors for the aerobic respiratory chain (15, 37–39). Through this hidden metabolic flexibility, organisms that require organic carbon for growth can still persist in dormant states in continually carbon-depleted soils (6, 40). The relative importance of these strategies may vary depending on the water availability of desert ecosystems: energy reserves may be more important in semiarid ecosystems, whereas trace gas oxidation may be the dominant energy conservation strategy in hyperarid deserts with minimal photosynthetic input (6, 41).

The role of inorganic compounds as alternative energy sources for desert communities has been particularly overlooked. Two recent studies showed that the dominant microorganisms in Antarctic desert soils are metabolically flexible bacteria capable of using both organic and inorganic energy sources, most notably atmospheric hydrogen. Through genome-resolved metagenomics, it was shown that bacteria from phyla such as *Actinobacteriota* encode specific high-affinity hydrogenases (group 1h and 1l [NiFe]-hydrogenases) to oxidize atmospheric H_2_. Activity studies have shown that electrons derived from H_2_ oxidation can support aerobic respiration and, for some cells capable of the Calvin-Benson-Bassham cycle, carbon dioxide fixation (15, 37). Based on this evidence, it has been inferred that these bacteria are capable of “living on air” (42). These findings are supported by pure culture studies that show high-affinity hydrogenases are most expressed in carbon-limited cells (43–47) and, when knocked out, result in decreased long-term survival (48–50). Diverse bacteria can also support mixotrophic growth using atmospheric H_2_ (51–54). Overall, atmospheric H_2_ is a highly dependable substrate for bacteria in oligotrophic environments given that it is ubiquitous in the lower atmosphere (mixing ratio, 0.53 ppmv), has a low activation energy, and yields a large amount of free energy when oxidized (55–58). Moreover, in contrast to water-soluble substrates, gaseous substrates readily diffuse through cell membranes and are more available to cells in drier soils as a result of diffusion through air-filled soil pores (7, 57, 59). Thus, atmospheric H_2_ oxidation may provide a minimalistic way for bacteria to sustain energy and carbon needs in otherwise energy-depleted desert environments.

Despite these advances, it remains unclear whether trace gas oxidation is also a key process in desert ecosystems outside Antarctica. Previous studies have identified hydrogenase genes in nonpolar desert soils, but their activity has not been confirmed (39, 60, 61). We also lack a systematic understanding of how this process is influenced by hydration and how it relates to other metabolic strategies such as photosynthesis. A recent study focused on the Colorado Plateau showed that, whereas hydrogenase-encoding transcripts were highly enriched in dry biocrusts, photosystems became more enriched following wetting; this suggests a possible shift from a chemosynthetically driven dryland community to a photosynthetically driven hydrated one (61). In this study, we addressed these knowledge gaps by investigating the role of trace gas oxidation relative to photosynthetic carbon fixation in wet and dry desert soils. To do so, we compared the distribution, expression, and activities of the enzymes responsible for atmospheric H_2_ oxidation and photosynthetic CO_2_ fixation. We compared profiles over the course of a simulated hydration-desiccation cycle through a microcosm experiment and generalized key findings to arid deserts sampled across four continents. We show that atmospheric H_2_ oxidizers are abundant in climatically diverse nonpolar deserts but, in contrast to Antarctic soil communities, had low levels of activity except when hydrated and likely adopt a mixotrophic lifestyle. Based on these findings, we readdress the two hypotheses for how chemoheterotrophs are sustained in desert ecosystems.

## RESULTS

### Uptake hydrogenases are encoded by chemoheterotrophic and photoautotrophic bacteria in an Australian desert.

**FIG 1:**
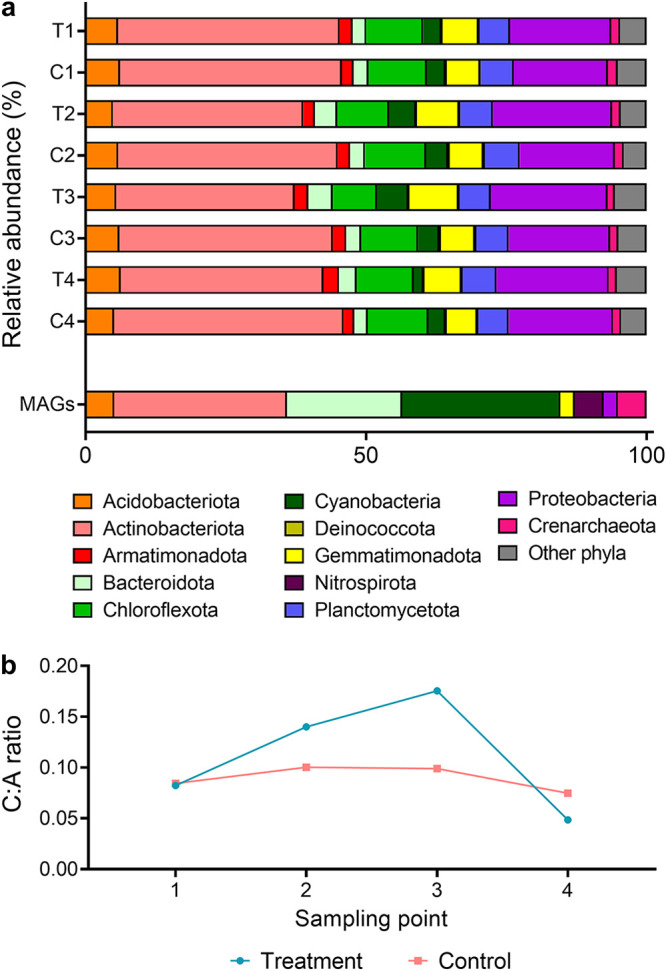
Changes in phylum-level community composition of the Australian desert soil microcosms during hydration and desiccation. (a) Stacked bar charts showing phylum-level community composition of the Australian desert soil microcosms. Metagenomes were sequenced from control microcosms (C; nonwatered) and treatment microcosms (T; watered) at four sampling points: 210 h (C1 and T1; 9 days after microcosm setup and 24 h before first wetting), 258 h (C2 and T2; 24 h after first wetting), 306 h (C3 and T3; 24 h after third wetting), and 1,002 h (C4 and T4; 30 days after third wetting). Community composition is based on metagenomic short reads using the GraftM pipeline. The proportion of the 39 metagenome-assembled genomes (MAGs) that were affiliated with each phylum is also shown. (b) Ratio of *Cyanobacteria* (C) to *Actinobacteriota* (A), based on relative abundance, at each sampling point.

Surface soils (0 to 10 cm depth) were collected from a sparsely vegetated arid desert in South Australia (see Table S1 in the supplemental material). Metagenomic profiling revealed that diverse bacteria and archaea coexisted in these soils in their dry native state (Fig. S4). In common with most desert soils (6), the most abundant community members were organoheterotrophic lineages within the phyla *Actinobacteriota* (39%), *Proteobacteria* (17%), and *Chloroflexota* (10%) (Fig. 1a). *Cyanobacteria* were also moderately abundant (3.2%), especially *Nostoc*, *Microcoleus*, and *Tychonema* taxa (Table S3), all of which have previously been described for desert biocrusts and soils (62–64). Metagenomic assembly yielded 39 metagenome-assembled genomes (MAGs) from eight phyla, including multiple genomes of *Actinobacteriota*, *Cyanobacteria*, and *Bacteroidota* (Fig. 1a; Table S2). However, despite high sequencing depth (563 million read pairs [Table S2]), for unclear reasons binning only captured a small and skewed proportion of the diverse overall community.

10.1128/mSystems.01131-20.7 TABLE S1 Soil physicochemical properties of the Australian desert soil microcosms at each sampling point. Download Table S1, XLSX file, 0.01 MB. Copyright © 2020 Jordaan et al. 2020 Jordaan et al. This content is distributed under the terms of the Creative Commons Attribution 4.0 International license.

10.1128/mSystems.01131-20.8 TABLE S2 Sequencing statistics for the metagenomes and metatranscriptomes and taxonomic information and metabolic classification of the 39 metagenome-assembled genomes (MAGs). Download Table S2, XLSX file, 0.02 MB. Copyright © 2020 Jordaan et al. 2020 Jordaan et al. This content is distributed under the terms of the Creative Commons Attribution 4.0 International license.

10.1128/mSystems.01131-20.9 TABLE S3 Bacterial and archaeal community composition of Australian desert soil microcosms and other desert samples based on metagenomic searches using GraftM. Download Table S3, XLSX file, 0.2 MB. Copyright © 2020 Jordaan et al. 2020 Jordaan et al. This content is distributed under the terms of the Creative Commons Attribution 4.0 International license.

**FIG 2:**
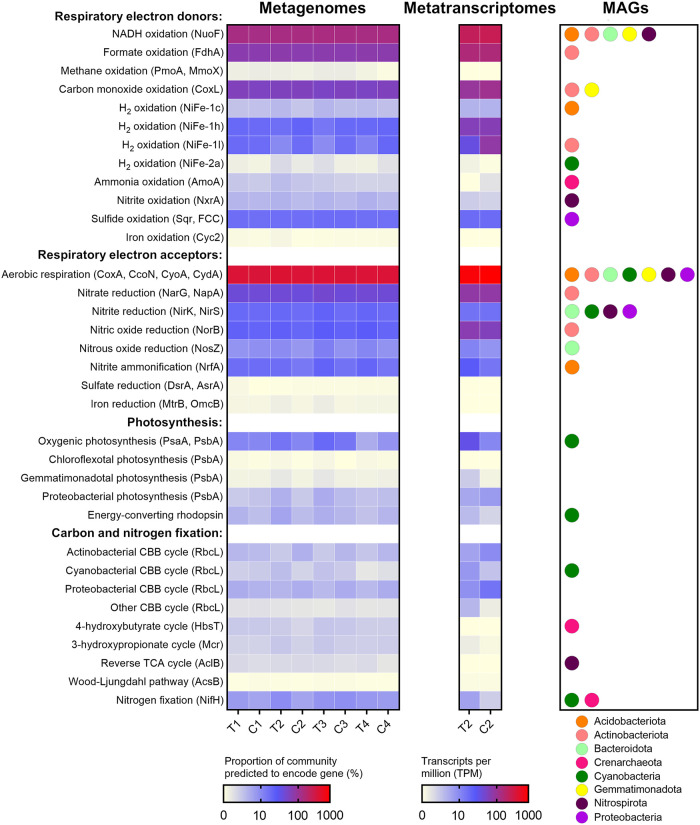
Abundance, expression, and distribution of metabolic marker genes in the Australian desert soil microcosms. The left heat map shows the proportion of the total community predicted to encode each gene, based on normalizing metagenomic reads for these genes to single-copy ribosomal marker genes, across the four sampling points. Where multiple marker genes are present per category, the abundances are summed. Where community proportion exceeds 100%, each community member is predicted to carry more than one gene per category on average (e.g., multiple types of cytochrome oxidase). The middle heat map shows the abundance of each gene in the mapped metatranscriptomic reads, based on transcripts per million (TPM), for the first postwetting sampling point. The right plot shows the bacterial and archaeal phyla known to have each gene in the microcosms, based on the 39 metagenome-assembled genomes (MAGs). Note that given the moderate number and completeness of the MAGs, the phylum-level distribution of the genes is not exhaustive, and as a result, some abundant genes in the metagenomic short reads are not represented by any MAGs (e.g., group 1h [NiFe]-hydrogenases, type ID and IE RuBisCO, and proteobacterial photosystem II). Marker genes encoding subunits of the following enzymes involved in respiration, photosynthesis, and carbon and nitrogen fixation are shown: NuoF (complex I), FdhA (formate dehydrogenase), PmoA (particulate methane monooxygenase), MmoX (soluble methane monooxygenase), CoxL (type I [MoCu]-carbon monoxide dehydrogenase), NiFe ([NiFe]-hydrogenases, groups 1c, 1h, 1l, and 2a), AmoA (ammonia monooxygenase), NxrA (nitrite oxidoreductase), Sqr (sulfide-quinone oxidoreductase), FCC (flavocytochrome *c* sulfide dehydrogenase), Cyc2 (iron-oxidizing cytochrome), CoxA (cytochrome *aa*_3_ oxidase), CcoN (cytochrome *cbb*_3_ oxidase), CyoA (cytochrome *bo*_3_ oxidase), CydA (cytochrome *bd* oxidase), NarG (dissimilatory nitrate reductase), NapA (periplasmic nitrate reductase), NirK (copper-containing nitrite reductase), NirS (cytochrome *bd*_1_ nitrite reductase), NorB (nitric oxide reductase), NosZ (nitrous oxide reductase), NrfA (ammonifying nitrite reductase), DsrA (dissimilatory sulfite reductase), AsrA (anaerobic sulfite reductase), MtrB (decaheme iron reductase), OmcB (iron-reducing cytochrome), PsbA (photosystem I), PsbA (photosystem II), energy-converting rhodopsins, RbcL (ribulose 1,5-bisphosphate carboxylase/oxygenase), HbsT (thaumarchaeotal 4-hydroxybutyryl coenzyme A [CoA] synthase), Mcr (malonyl-CoA reductase), AclB (ATP-citrate lyase), AcsB (acetyl-CoA synthase), and NifH (nitrogenase).

To resolve the metabolic capabilities of the community, we performed homology-based searches of the metagenomic short reads, assemblies, and MAGs against custom metabolic marker databases (50, 65, 66). As expected from the community profile (Fig. 1a), most community members encoded the enzymes for aerobic organotrophic respiration (e.g., NADH dehydrogenases and terminal oxidases) (Fig. 2). Many bacteria were also predicted to gain energy from oxidizing the inorganic compounds hydrogen (via uptake [NiFe]-hydrogenases [45% of total community]), carbon monoxide (via [MoCu]-CO dehydrogenases [57%]), and sulfide (via sulfide-quinone oxidoreductases and flavocytochrome *c* sulfide dehydrogenases [16%]), including MAGs affiliated with *Actinobacteriota*, *Proteobacteria*, and other abundant phyla (Fig. 2; Table S2). Marker genes of ammonia-oxidizing archaea (ammonia monooxygenases [1.9%]) and nitrite-oxidizing bacteria (nitrite oxidoreductases [3.1%]) were also present. While most anaerobic respiratory pathways were in low abundance, diverse community members encoded enzymes to mediate steps of denitrification and dissimilatory nitrate reduction to ammonium pathways (Fig. 2). Light harvesting and carbon fixation genes were encoded by *Cyanobacteria* as expected but also, to a lesser extent, by other taxa in line with the continual energy-harvesting hypothesis (6). These include short reads and assembled sequences homologous to the anoxygenic photosystems of *Proteobacteria*, *Chloroflexota*, and *Gemmatimonadota*, as well as various energy-converting rhodopsins (Fig. 2; Tables S2 and S4). We therefore infer that light simultaneously supports photoautotrophic growth of *Cyanobacteria* and serves as an energy input for a low number of photoheterotrophic taxa in this community. A total of 9% of community members carried RuBisCO genes to fix carbon dioxide through the Calvin-Benson-Bassham cycle. Phylogenetic analysis confirmed that most RuBisCO sequences were closely related to reference sequences from *Cyanobacteria* (type IB), *Actinobacteriota* (type IE), and *Proteobacteria* (types IA, IC, and ID) (Fig. S5). Also detected were the determinants of the 4-hydroxybutyrate cycle (MAGs of ammonia-oxidizing *Crenarchaeota*), reverse tricarboxylic acid cycle (MAGs of nitrite-oxidizing *Nitrospirota*), and 3-hydroxypropionate cycle (short reads matching *Chloroflexota* reference sequences) (Fig. 2; Table S2 and S4). This suggests that, in common with polar desert ecosystems (15, 37), this hot desert harbors a substantial capacity for chemosynthetic carbon fixation.

10.1128/mSystems.01131-20.10 TABLE S4 Metabolic marker gene abundance in metagenomic and metatranscriptomic short reads and sequences of their hits in assemblies and MAGs. Download Table S4, XLSX file, 2.8 MB. Copyright © 2020 Jordaan et al. 2020 Jordaan et al. This content is distributed under the terms of the Creative Commons Attribution 4.0 International license.

To resolve the mediators and role of H_2_ oxidation in this ecosystem, we classified sequences encoding hydrogenase catalytic subunits using the recently expanded HydDB scheme (15, 66). The most abundant lineages were the high-affinity group 1h and 1l [NiFe]-hydrogenases (Fig. 2; Table S4), which support persistence by atmospheric H_2_ oxidation in *Actinobacteriota*, *Chloroflexota*, and other lineages (15, 44, 46–48). Phylogenetic analysis revealed that these hydrogenases formed diverse radiations (Fig. S6), including clusters closely related to reference genomes of the five most abundant orders of *Actinobacteriota* and *Chloroflexota* present in the community (Table S3). Also present were [NiFe]-hydrogenase subgroups affiliated with *Acidobacteriota* (group 1c), *Proteobacteria* (group 1d), and *Cyanobacteria* (group 2a) (Fig. S6). The low-affinity group 1d [NiFe]-hydrogenases and medium-affinity group 2a [NiFe]-hydrogenases have dual roles in recycling endogenously produced H_2_ and supporting hydrogenotrophic growth on exogenous H_2_ (51, 67, 68), whereas the function of group 1c hydrogenases in aerobes is unresolved (58). Concordantly, group 2a [NiFe]-hydrogenases were present in three cyanobacterial MAGs, namely, the nitrogen-fixing filamentous *Nostoc* and *Tolypothrix* (Fig. 2). While H_2_ oxidation in aquatic cyanobacteria is well known (69, 70), to our knowledge this is the first report of desert cyanobacteria harboring uptake hydrogenases. In energy-deprived desert soils, a key role of this hydrogenase is likely to recycle H_2_ endogenously produced by the energetically demanding nitrogenase reaction, as previously reported for other *Nostoc* strains (71, 72). However, given recent discoveries regarding the physiological role of group 2a [NiFe]-hydrogenase (51), it is also plausible that this hydrogenase enables these bacteria to conserve energy by scavenging exogenous H_2_. Together, these genomic inferences provide considerable support for the continual energy-harvesting hypothesis by uncovering flexibility in energy conservation and carbon acquisition pathways, including hydrogenases associated with the most abundant chemoheterotrophic and photoautotrophic taxa.

### Hydration and desiccation cause moderate changes in community composition and metabolic gene expression in desert microorganisms.

A microcosm experiment was performed to investigate how community and metabolic composition, gene expression, and biogeochemical activities of the sampled Australian soils (Fig. S1) varied during a simulated hydration-desiccation cycle over a 41-day period (Fig. S2). Relative humidity monitoring using iButtons showed the duration that water treatment microcosms were fully saturated as a result of three water pulses before returning to dry state (Fig. S3). The unwetted control microcosms also became more humid, given that they were stored in the same incubator in order to reduce extraneous effects, though below the threshold to cause major detectable changes in physicochemical properties (Table S1), metagenomic profiles (Fig. 1 and 2), or biogeochemical activities.

10.1128/mSystems.01131-20.1 FIG S1 Geographical details of the samples studied. (a) Map showing location of four study sites colored by Koppen-Geiger Climate Classification. (b) Photograph of the exact sampling site used for the microcosm experiments near Roxby Downs, South Australia. Download FIG S1, TIF file, 1.1 MB. Copyright © 2020 Jordaan et al. 2020 Jordaan et al. This content is distributed under the terms of the Creative Commons Attribution 4.0 International license.

10.1128/mSystems.01131-20.2 FIG S2 Overview of study design and setup of the Australian desert soil microcosms for the control samples (a) and water treatment samples (b). Download FIG S2, TIF file, 2.3 MB. Copyright © 2020 Jordaan et al. 2020 Jordaan et al. This content is distributed under the terms of the Creative Commons Attribution 4.0 International license.

10.1128/mSystems.01131-20.3 FIG S3 Temperature and humidity profiles of the Australian desert soil microcosms. Temperature and humidity were monitored by iButtons embedded in each microcosm, with measurements taken every 20 min over a 44-day period. The traces show the average hydration and temperature across the five independent control microcosms and five independent treatment microcosms. The three wetting time points are depicted by the green dotted lines, and the four sampling points are depicted by red dashed lines. Download FIG S3, TIF file, 0.4 MB. Copyright © 2020 Jordaan et al. 2020 Jordaan et al. This content is distributed under the terms of the Creative Commons Attribution 4.0 International license.

For the treatment microcosms, hydration resulted in minor but important changes at the community level. Based on composite metagenomes, alpha diversity did not substantially change (Fig. S4), though modest changes in microbial composition were observed (Fig. 1a). Notable changes included an increase in relative abundance (up to 1.7-fold) of lineages capable of oxygenic photosynthesis (e.g., *Nostocales*) and anoxygenic photosynthesis (e.g., *Rhodobacterales*), concomitant with a decrease of similar magnitude of two actinobacterial orders (*Rubrobacterales* and *Gaiellales*). This trend was reversed as soils returned to a desiccated state at the end of the experiment (Fig. 1a; Table S3). This is reflected by the shifts in the ratios of *Cyanobacteria* to *Actinobacteriota* during the time course in the composite metagenomes (Fig. 1b). The community composition of the control microcosms, in contrast, was stable over the 41 days (Fig. 1). Based on these composite metagenomes, the abundances of *Cyanobacteria*-associated genes, such as for photosystems I and II, type IB RuBisCO, and group 2a [NiFe]-hydrogenase, were at least 1.5-fold higher in the metagenomes of the hydrated microcosms (T2 and T3) than for controls (C2 and C3), whereas the abundance of *Actinobacteriota*-associated group 1l [NiFe]-hydrogenase and type IE RuBisCO decreased to an even greater extent (Fig. 2). Our findings contrast with some studies that have reported major shifts following wetting, including community shifts from aerobes to anaerobes (24, 73). This may reflect differences in microcosm setup and wetting intensity, the latter designed to simulate natural precipitation pulses rather than flooding events. Together, these findings substantiate that hydration modulates desert communities from being dominated by dormant chemoheterotrophs to containing significant levels of actively growing photoautotrophs.

10.1128/mSystems.01131-20.4 FIG S4 Alpha diversity of Australian desert soil microcosms. Values are based on the single-copy ribosomal marker gene *rplB* retrieved from metagenomic reads. (a) Estimated richness based on Chao1 index. (b) Taxon diversity based on Shannon index. Download FIG S4, TIF file, 0.3 MB. Copyright © 2020 Jordaan et al. 2020 Jordaan et al. This content is distributed under the terms of the Creative Commons Attribution 4.0 International license.

10.1128/mSystems.01131-20.5 FIG S5 Phylogenetic tree of amino acid sequences of ribulose 1,5-bisphosphate carboxylase/oxygenase (RuBisCO) catalytic subunits. Sequences are shown of binned (red) and unbinned assembled (blue) sequences retrieved from the metagenomes alongside representative reference sequences (gray). The RuBisCO subgroup associated with each reference sequence is shown, with type IB associated with photoautotrophs and the retrieved type IA, IC, ID, and IE being associated with chemoautotrophs. Trees were constructed by the neighbor-joining method, gaps were treated with partial deletion, and bootstrapping used 200 replicates. Download FIG S5, PDF file, 0.1 MB. Copyright © 2020 Jordaan et al. 2020 Jordaan et al. This content is distributed under the terms of the Creative Commons Attribution 4.0 International license.

10.1128/mSystems.01131-20.6 FIG S6 Phylogenetic tree of amino acid sequences of group 1 and 2 [NiFe]-hydrogenase catalytic subunits. Sequences are shown of binned (red) and unbinned assembled (blue) sequences retrieved from the metagenomes alongside representative reference sequences (gray). The hydrogenase subgroup associated with each reference sequence is shown, with group 1h, 1l, 2a, and 1f [NiFe]-hydrogenases previously shown to mediate atmospheric H_2_ oxidation. Trees were constructed by the neighbor-joining method, gaps were treated with partial deletion, and bootstrapping used 200 replicates. Download FIG S6, PDF file, 0.02 MB. Copyright © 2020 Jordaan et al. 2020 Jordaan et al. This content is distributed under the terms of the Creative Commons Attribution 4.0 International license.

To gain further insight into community responses to hydration, we sequenced and mapped metatranscriptomes of composite wet treatment microcosms following the first hydration event (T2) and compared them to the metatranscriptomes of composite dry control microcosms (C2). The most expressed metabolic genes surveyed in the dry condition were the standard complexes of the respiratory chain (AtpA, NuoF, SdhA, and CoxA; all >200 transcripts per million [TPM]) followed by group 1h and 1l [NiFe]-hydrogenases (144 TPM), formate dehydrogenase (FdhA; 141 TPM), and carbon monoxide dehydrogenase (CoxL; 105 TPM) (Fig. 2; Table S4). The high expression of these genes highlights that most community members adapt to dry conditions in deserts through a mixotrophic lifestyle, combining respiration of exogenous organic compounds and macromolecular reserves with oxidation of atmospheric H_2_ and carbon monoxide. While these findings lend support for continual energy harvesting from trace gases, fewer community members expressed genes to harvest light energy (photosystems of anoxygenic photosynthesis, 6.3 TPM; energy-converting rhodopsins, 1.3 TPM). As expected, the levels of cyanobacterial photosystem I (5.1 TPM), photosystem II (4.1 TPM), and RuBisCO (2.2 TPM) were also low (Fig. 2), in line with expectations that water scarcity limits oxygenic photosynthesis. In contrast, there was moderate expression of chemosynthetic RuBisCO lineages (23 TPM) (Fig. 2), suggesting some basal chemoautotrophic primary production in common with Antarctic soils (37).

Following hydration, the expression of most of the above-mentioned respiratory genes remained high. However, when normalized to whole-community expression, there was a 3-fold increase in the relative expression of photosystems and RuBisCO genes affiliated with *Cyanobacteria*, together with a 1.7-fold decrease in chemosynthetic RuBisCO lineages (Fig. 2). This suggests a shift from chemoautotrophic to photoautotrophic primary production, in line with our predictions (41). More surprisingly given previous observations (61), hydrogenase gene expression remained very high (Fig. 2), and thus, H_2_ oxidation is likely to be a key process even in hydrated soils; specifically, expression of [NiFe]-hydrogenases from groups 1h and 1c remained stable, whereas those from group 2a increased and those from group 1l decreased, in line with observations from the metagenome-based community and metabolic profiles (Fig. 2; Table S4). The composite metatranscriptomes also confirmed moderate expression of genes for denitrification, sulfide oxidation, and, following hydration, nitrogen fixation. As we sequenced nucleic acid extracted from composite rather than replicate microcosms, it is not possible to perform statistical tests on how gene abundance and expression changed during the simulated hydration-desiccation cycle. In addition, it should be noted that transcription levels are likely to be much higher overall following wetting and hence mild variations in relative expression levels (i.e., TPM) may translate to large increases in protein synthesis and enzymatic activities (6).

### Hydrogen oxidation and carbon fixation activities are greatly stimulated by hydration in desert soils.

**FIG 3:**
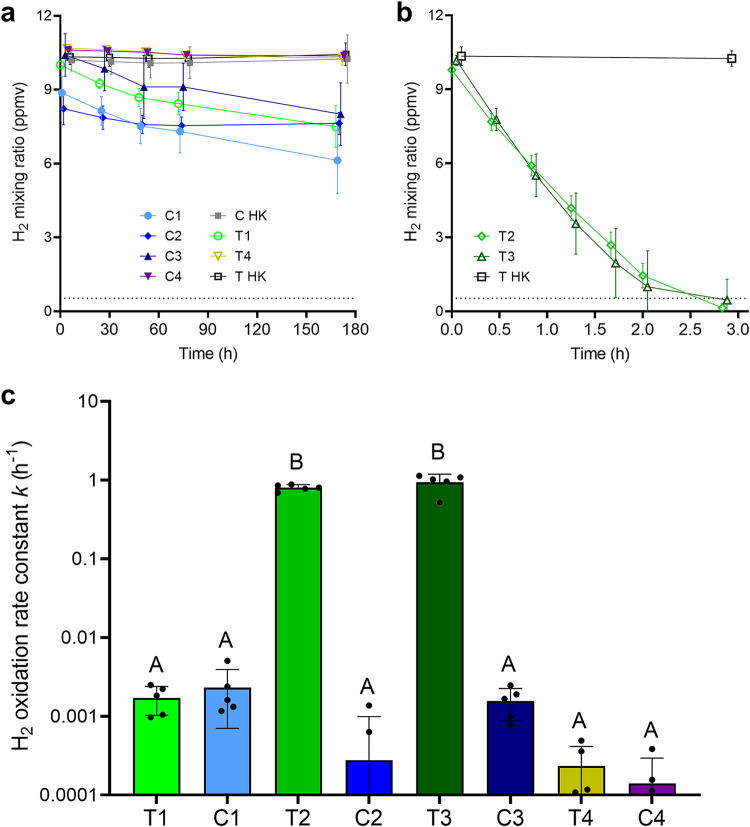
H_2_ oxidation by Australian desert soil microcosm samples. Headspace H_2_ mixing ratios were measured by gas chromatography over an extended period (180 h) for slow-oxidizing dry samples (T1, T4, C1, C2, C3, and C4) (a) and a short period (3 h) for fast-oxidizing wet samples (T2 and T3) (b). Symbols show means and error bars show standard deviations from five independent microcosms. Heat-killed controls were also tested for each sampling point (C HK and T HK). The gray dashed line at 0.53 ppmv indicates the average global atmospheric concentration of H_2_. (c) Comparison of first-order rate constants (*k*) for H_2_ oxidation calculated at each sampling point based on five replicate microcosms. Samples labeled with different letters are significantly different based on one-way ANOVA (*P < *0.0001). As heat-killed controls did not consume H_2_, rate constants are shown only for the samples with live microorganisms.

Together, the metagenomic and metatranscriptomic analyses suggest that H_2_ is a major energy source for the Australian desert soil samples in both dry and wet states. To test this, we used gas chromatography to monitor H_2_ consumption of the soils incubated in an ambient air headspace supplemented with ∼10 ppmv. In accord with an enzymatic process, H_2_ oxidation occurred with first-order reaction kinetics in live soils but was not detected in heat-killed soils. While measurable H_2_ uptake was observed in the native dry soils (Fig. 3a), activities were low (first-order rate constant *k* = 0.0020 ± 0.012 h^−1^) (Fig. 3c), and oxidation continued even after one month of incubation. These findings are contrary to observations in Antarctic soils, which mediate rapid H_2_ oxidation even in their native state (15, 37). However, they are in line with other reports that microbial H_2_ uptake ceases at very low moisture content for as-yet-unresolved reasons (74). These rates are unlikely to be sufficient to sustain the maintenance needs of community members (75, 76), unless cells have ultralow requirements as recently described for aerobic heterotrophs in other energy-deprived environments (77). Instead, it is likely that hydrogenase-expressing community members supplement energy derived from H_2_ with other sources (e.g., organic compounds, carbon monoxide, formate, and light) to survive in these soils. Remarkably, hydration caused a significant 950-fold increase (*P < *0.0001) in the rate of H_2_ oxidation of the treatment microcosms at the second and third sampling points (*k* = 0.87 ± 0.19 s^−1^) compared to the control microcosms (Fig. 3c). The communities consumed H_2_ to subatmospheric levels down to the quantification limit (61 ppbv) within 3 h (Fig. 3b). This supports data indicating the high abundance and expression of hydrogenases in the community even postwetting. The simulated desiccation event caused a decline in H_2_ oxidation activity to levels approaching the limit of detection (Fig. 3a). A highly consistent response was observed across all five microcosm replicates per condition, indicating that our inferences based on pooled metagenomes and metatranscriptomes are not unreasonably skewed by intermicrocosm variability.

**FIG 4:**
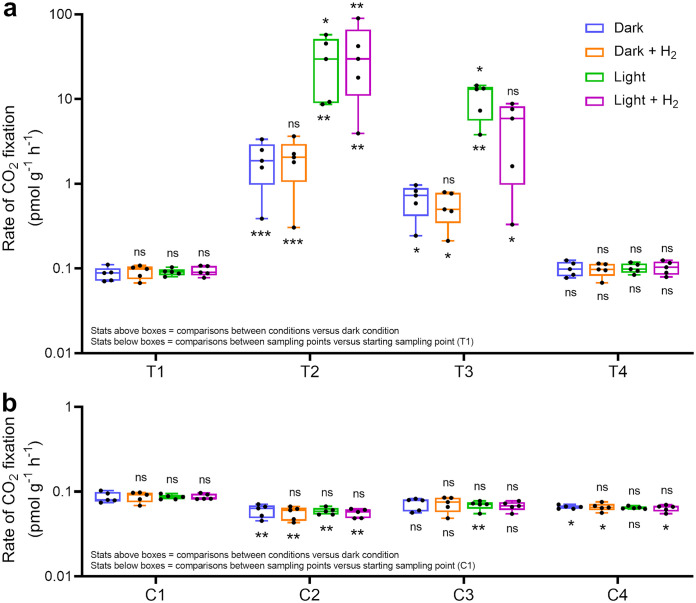
Carbon dioxide fixation by the Australian desert soil microcosm samples. Samples from treatment microcosms (a) and control microcosms (b) were incubated with ^14^C-labeled CO_2_ under four different conditions. Rates were determined by counting ^14^C fixed in live samples and subtracting background counts from heat-killed controls. Each individual boxplot shows the rate of ^14^C fixation for five independent microcosms each run in technical quadruplicate (averaged). Center values show medians, boxes show upper and lower quartiles, and whiskers show maximum and minimum values. Symbols above the boxes indicate whether significant differences were observed between H_2_-supplemented, light-illuminated, and joint treatment conditions relative to the dark condition at each sampling point. One-way ANOVA was used to test significant differences. ns, not significant. *, *P < *0.05; **, *P < *0.01; ***, *P < *0.001. Symbols below the boxes indicate whether significant differences were observed between the sampling points for each condition relative to the starting sampling point (T1 and C1). Significant differences were also observed in ^14^C counts between the T2 and C2 samples (*P < *0.01), and the T3 and C3 samples (*P < *0.05), under each condition.

We also traced the incorporation of radiolabeled carbon dioxide (^14^CO_2_) to compare the rates of chemosynthetic and photosynthetic carbon fixation during the microcosm experiment. CO_2_ incorporation occurred at low but detectable rates in the native dry soils compared to the heat-killed controls (0.089 ± 0.011 pmol g^−1^ h^−1^), though it was not significantly affected by the availability of light and/or H_2_ (Fig. 4). Hydration caused a 20-fold stimulation (*P = *0.004, one-way analysis of variance [ANOVA]) in dark CO_2_ assimilation rates for the treatment microcosms at the second and third sampling points (1.30 ± 0.99 pmol g^−1^ h^−1^) (Fig. 4a) compared to the control microcosms (0.066 ± 0.012 pmol g^−1^ h^−1^) (Fig. 4b). Light illumination caused a further 16-fold increase (*P = *0.008, one-way ANOVA) in CO_2_ fixation compared to the dark condition for the treatment microcosms, but not the controls (Fig. 4b). In line with the increased abundance and transcriptional activity of cyanobacteria (Fig. 1 and 2), this confirms that hydration stimulates oxygenic photosynthesis in hot desert soils as expected. However, unlike for Antarctic soils (37), H_2_ supplementation did not significantly stimulate CO_2_ fixation, and hence H_2_-oxidizing bacteria are unlikely to be major primary producers in these wet soils. Instead, dark assimilation may reflect chemoautotrophic activity of ammonia-, nitrite-, or sulfide-oxidizing microorganisms that are present in the community (Fig. 1) and transcriptionally active to various extents (Fig. 2), though anaplerotic CO_2_ incorporation cannot be ruled out. Together, the expression and activity profiles suggest that photoautotrophs are the major primary producers in these desert soils when hydrated. In contrast, H_2_ is primarily used as an electron donor for respiration rather than carbon fixation.

### Atmospheric H_2_ oxidizers are abundant community members in three other deserts and are stimulated by hydration.

**FIG 5:**
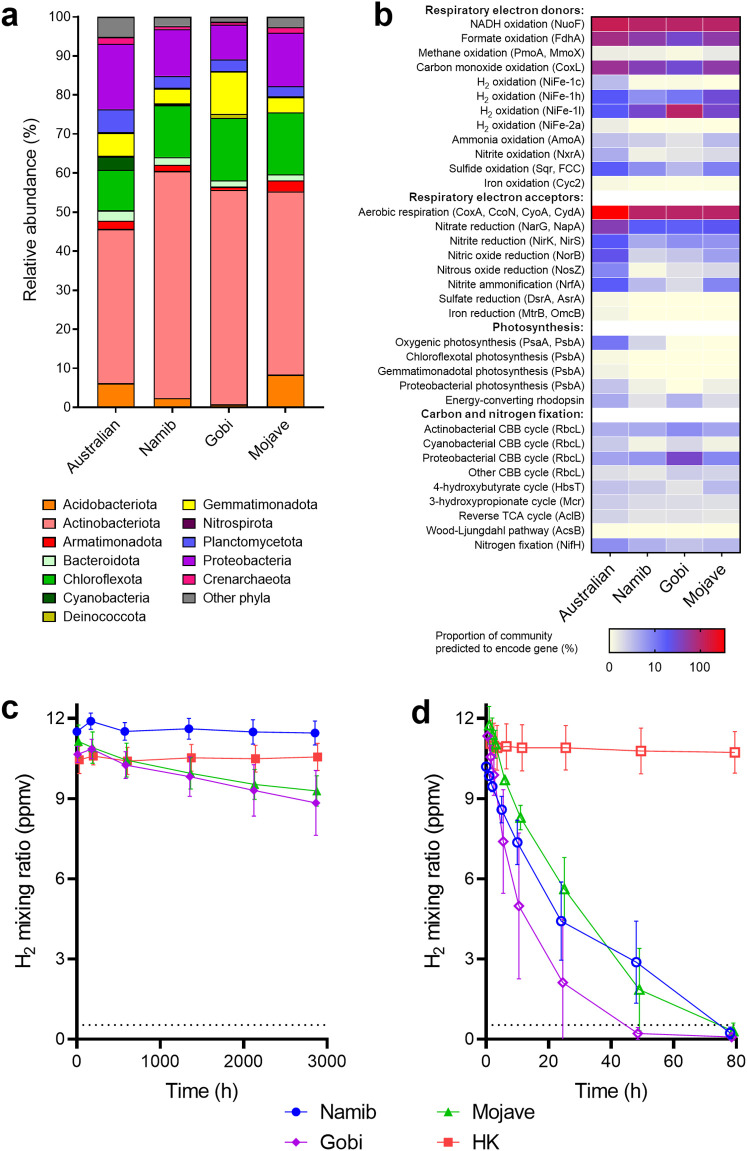
Community composition, metabolic potential, and H_2_ oxidation activities of three additional desert soils. Results are shown for surface soils collected from the Namib, Gobi, and Mojave deserts. (a) Stacked bar charts show phylum-level community composition based on metagenomic short reads using the GraftM pipeline. (b) Proportion of total community predicted to encode marker genes for respiration, photosynthesis, and carbon and nitrogen fixation, based on normalizing metagenomic reads for these genes to single-copy ribosomal marker genes. Community and metabolic profiles of the Australian desert soil, based on the C1 metagenome, are shown for comparison. H_2_ oxidation of native dry samples (c) and experimentally wetted samples (incubated 1 day postwetting) (d) relative to heat-killed (HK) controls was measured by gas chromatography. Symbols show means and error bars show standard deviations from three independent samples. The gray dashed line at 0.53 ppmv indicates the average global atmospheric concentration of H_2_.

To test whether these findings were generalizable to deserts in other continents, we sampled arid soils from the climatically distinct Namib, Gobi, and Mojave deserts. Metagenomic profiling showed that the community composition of these soils was relatively similar to those of the Australian microcosms at the phylum and order levels (Table S4). However, Actinobacteriota were more abundant (47% to 58%), whereas *Cyanobacteria* were scarce (0 to 0.3%) (Fig. 5a; Table S3). As for Australian samples, most community members were predicted to be capable of aerobic organotrophic respiration. In further support of the continual energy-harvesting hypothesis, the genes for hydrogen, carbon monoxide, formate, and, to a lesser extent, sulfide oxidation were widely distributed, whereas photosystem and rhodopsin genes were scarce (Fig. 5b). Particularly notable is the observation that almost all community members in the Gobi desert are predicted to encode the group 1l [NiFe]-hydrogenase, in common with soils recently described for the Mackay Glacier region of South Victoria Land, Antarctica (15). Activity measurements confirmed that H_2_ consumption was very slow in the native dry soils (Fig. 5c) but was rapidly stimulated by hydration (1,031-fold, *P = *0.011, Student's *t* test) and occurred to subatmospheric levels (Fig. 5d). This suggests that, in common with the Australian desert soils, the microorganisms in these deserts adopt a mixotrophic strategy to persist and rapidly mobilize atmospheric H_2_ alongside likely other resources when hydration levels are sufficient. Thus, we observed across four distinct deserts (Australian, Mojave, Gobi, Namib) that hydrogen-oxidizing bacteria are abundant community members that are strongly stimulated by hydration, demonstrating the generalizability of our core conclusions.

## DISCUSSION

This study suggests that multiple energy sources, including atmospheric H_2_, are important for the endurance of microbial communities in desert environments. We previously postulated that the relative importance of photoautotrophs (central to the energy reserve hypothesis) and trace gas oxidizers (central to the continual energy-harvesting hypothesis) would shift depending on hydration levels (6, 41). We obtained some evidence for opposing responses of these functional groups to hydration, as well as a much higher genomic potential for trace gas oxidation than for photoautotrophy and photoheterotrophy overall. However, the metagenomic, metatranscriptomic, and biogeochemical data suggest that atmospheric H_2_ oxidation and photosynthetic carbon fixation are both highly active in wetted soils. To survive in these resource-poor environments, dominant community members such as *Actinobacteriota*, *Proteobacteria*, and *Acidobacteria* are likely to simultaneously use endogenous and exogenous organic carbon generated during hydration events alongside dependable exogenous substrates such as trace gases. This mixotrophic strategy may help cells rapidly replenish energy and carbon amid intense competition following hydration events. In turn, these findings add to growing evidence for the importance of metabolic flexibility and inorganic energy sources in sustaining life in deserts and other extreme soil environments (6, 15, 37, 39, 78).

In accord with conventional ecological theory, our study highlights that *Cyanobacteria* are the most active autotrophs in hydrated soils. While metagenomic signatures of lithoautotrophy were detected in all four desert samples, based on the carbon fixation assays performed only on the Australian desert samples, there was considerable dark carbon assimilation but no significant hydrogenotrophic carbon fixation in dry and wet soils. This suggests that most H_2_-oxidizing bacteria use electrons derived from trace gases to support aerobic respiration, rather than the energetically demanding process of carbon fixation. It is plausible that hydrogenotrophic carbon fixation is a more dominant process in hyperarid soils, where there is minimal nutrient input from photoautotrophs; this is consistent with the higher relative abundance of facultative lithoautotrophs in microbial communities within Antarctic and Atacama desert soils, as well as the rapid carbon fixation measured in native dry soils in two Antarctic regions (15, 37, 39). With respect to primary producers, it is notable that some desert *Cyanobacteria* also have the capacity to use H_2_ as an alternative energy source, suggesting that even apparently obligate photoautotrophs benefit from metabolic flexibility to meet energy needs in this environment. However, further culture-based studies are required to determine whether uptake hydrogenases in desert *Cyanobacteria* primarily serve to recycle endogenous nitrogenase-derived H_2_ (i.e., enhancing energetic efficiency) or consume exogenous atmospheric H_2_ (i.e., broadening the energetic repertoire). In addition, further studies are required to gain a more detailed understanding of carbon fixation and energy acquisition strategies at the population and cellular levels.

A further surprising observation was that, despite the genes being highly expressed, atmospheric H_2_ oxidation occurred at low rates in dry soils and was greatly stimulated by hydration. This suggests that low moisture content greatly inhibits atmospheric H_2_ oxidation, though likely not as much as other processes such as photosynthetic carbon fixation. It seems improbable that the low rates of H_2_ oxidation measured under dry conditions are sufficient to sustain the large community of H_2_-oxidizing bacteria detected by metagenomics. However, given reports of extremely low energy requirements of dormant bacteria (77) and that some desiccation-tolerant genera can resuscitate following extensive macromolecular damage (79), it is possible that hot desert bacteria require extraordinarily low energetic input to survive in this environment. Further studies are required to determine the maintenance energies of bacteria in desiccated desert soils and the relative contributions of atmospheric H_2_ oxidation and other processes (e.g., macromolecular reserve degradation, relic organic carbon oxidation, carbon monoxide scavenging, and light harvesting) in sustaining them. It also remains to be explained why, despite harboring a similar relative abundance of predicted H_2_-oxidizing cells, atmospheric H_2_ oxidation in dry native samples occurred at much lower rates in the four desert soils described here than in previously reported Antarctic samples (15, 37). Further theoretical and experimental work should explore the physicochemical and biological factors driving differences in energy conservation and carbon fixation processes between polar and nonpolar deserts.

## MATERIALS AND METHODS

### Soil collection and processing.

Soils for the microcosm experiments were collected from an arid site (coordinates, −30.470422, 137.136334; elevation, 99 m) on the outskirts of Roxby Downs, South Australia, on 31 October and 1 November 2018. As depicted in the photograph in Fig. S1, the landscape is characterized by red sandy surface soil (100 to 200 mm thick) with gibber surface rocks covering clay and clay loam subsoil. Biological soil crusts and sparse vegetation (chenopod shrubland) were present. The sampling region has an average annual temperature of 27.8°C and mean annual precipitation of ∼145 mm, largely driven by occasional large rainfall events during summer months and occasional smaller pulses (www.bom.gov.au). In order to limit sampling bias and to ensure sampling homogeneity, the site was divided into three 10-m^2^ sections (located ∼120 m apart) and further subdivided into three 1-m^2^ subplots. Nine surface soil samples (0 to 10 cm) were aseptically collected and transferred into sterile 25-liter plastic containers. To create a representative sample for the complete site, the nine individual subsamples were pooled and homogenized by sieving through sterilized 2-mm grid size sieves. Samples were transported to the laboratory and stored at ambient temperature until microcosm setup 1 week after collection. Samples were also separately collected from three other arid deserts **(**Fig. S1**)**: a gravel plain (0 to 10 cm) from the Namib Desert, Namibia (coordinates, −23.31069, 15.53458; elevation, 946 m; 12 April 2016), a soil surface (0 to 2 cm) from the Gobi Desert, China (coordinates 39.902172, 96.226681; elevation, 2,514 m; 11 July 2018), and a loess pediment (0 to 10 cm) from the Mojave Desert, Nevada (coordinates, 35.79368, −114.86253; elevation, 846 m; 26 March 2019). In all cases, samples were aseptically collected and transferred into zipper storage bags before transport to Monash University’s quarantine approved facilities.

### Microcosm setup and sampling.

The Australian desert samples were subjected to a microcosm experiment that simulated a hydration-desiccation cycle. A total of 40 microcosms (20 for control, C; 20 for water treatment, T) were constructed (Fig. S2). These comprised open-top Plexiglas G-UVT acrylic vessels (5.5-cm height, 10-cm diameter) with a base made of a fine stainless-steel mesh screen. The vessels were cleaned with 80% (vol/vol) ethanol and sterilized with UV light for 30 min prior to setup. A total of 350 g of composite soil in its native dry state was added to each vessel. Microcosms were maintained at an average temperature of 28 ± 0.2°C (corresponding to the annual mean maximum temperature in Roxby Downs) in a Panasonic MLR 352H-PE climate control cabinet (Panasonic Healthcare Co., Ltd., Sakata, Japan). Light intensity was set at 20,000 lx (bulb type: FL40SS W/37) with a 12-h light, 12-h dark cycle. Soil temperature and relative humidity readings were recorded at 10-min intervals for 41 days using Hygrochron iButton temperature and humidity loggers (Maxim Integrated) (Fig. S3). These were placed at a depth of ∼2 cm in the center of each vessel. Soils were first allowed to stabilize in the vessels for 10 days prior to the beginning of the experiment. For the water treatment group, microcosms were watered with ultrapure water on three successive days to simulate a hydration event: day 10 (234 h; 70 ml), day 11 (258 h, 35 ml), and day 12 (282 h, 35 ml). The applied volume of water corresponds to heavy (∼6 mm h^−1^) and moderate (∼3 mm h^−1^) rainfall events in the sampled region and was predetermined through a trial experiment to prevent excess drainage of water and nutrients from soil microcosms. Microcosms were then left to dry for 31 days to simulate a desiccation event. Microcosms were destructively sampled at four points: day 9 (210 h; 24 h before wetting), day 11 (258 h; 24 h after first wetting), day 13 (306 h; 24 h after third wetting), and day 42 (1,002 h; 31 days after third wetting). At each sampling point, the contents of five control microcosms and five treatment microcosms were used for downstream soil physicochemical analysis, nucleic acid extraction, gas chromatography analysis, and carbon fixation assays.

### Soil physicochemical analysis.

Physicochemical analysis of Australian soil samples was conducted at the Environmental Analysis Laboratory (EAL), Southern Cross University, Australia, in accordance with ISO/IEC 17025 standard procedures. Composite soil samples were created for each sampling point and treatment group prior to analysis (Table S1). Physicochemical parameters analyzed included pH and electrical conductivity (1:5 water); total carbon, nitrogen, organic carbon, and organic matter; available calcium, magnesium, potassium, ammonium, nitrate, phosphate, and sulfur; exchangeable sodium, potassium, calcium, magnesium, hydrogen, and aluminum; and available micronutrients zinc, manganese, iron, copper, boron, and silicon. These data are summarized in Table S1. Chemical parameters were calculated following the methods of Rayment and Lyons (80).

### Metagenomic extraction and sequencing.

Metagenomic DNA was extracted from 40 soil samples collected during the Australian desert soil microcosm experiment (5 replicate microcosms × 2 treatment groups × 4 sampling points). DNA was extracted using 0.25 g of soil with the DNeasy PowerSoil kit (Qiagen) according to the manufacturer’s instructions. DNA purity and concentration were evaluated on an ND-1000 spectrophotometer (Thermo Fisher Scientific). DNA extracts for all soil replicates were pooled (equimolar basis) to generate a composite sample for each combination of sampling points and treatment group (i.e., eight composite samples: C1 to C4 and T1 to T4). Samples were stored at –80°C. Community genomic DNA (gDNA) samples were subjected to library preparation using the Nextera Flex DNA library prep kit followed by paired-end 150-bp sequencing on the Illumina HiSeq2500 platform at CD Genomics (New York). This yielded an average of 27.3 million paired reads per sample, as detailed in Table S2. Community DNA from the native dry Namib, Gobi, and Mojave desert samples were extracted using the same protocol and sequenced on a 2 × 150-bp NextSeq500 run at the Australian Centre for Ecogenomics, yielding an average of 15.4 million reads per sample (Table S2).

### Metatranscriptomic extraction and sequencing.

Total RNA was extracted using the method of León-Sobrino et al. (19), with minor modifications. At 24 h following the first wetting event, 2-g soil samples from each of the control microcosms (five C2 samples) and treatment microcosms (five T2 samples) were collected and preserved in RNAlater solution. The samples were washed in 10 ml of buffer containing 10 mM Tris-HCl, 1 mM EDTA, and 100 mM NaH_2_PO_4_ (pH 6.5) to remove excess salts. The pellets were mixed with 0.5 g of glass beads (equal volumes of 0.1-mm and 0.5-mm beads) and resuspended in 1 ml of lysis buffer (5% cetyltrimethylammonium bromide [CTAB], 0.7 M NaCl, 240 mM KH_2_PO_4_ [pH 8]) and 1 ml of TRIzol reagent (Invitrogen, Thermo Fisher Scientific). Samples were vortexed at 3,200 rpm for 1 min at room temperature, followed by chloroform (200 μl) and isopropanol (500 μl) washes. RNA was precipitated overnight at –20°C using 20 μl of 3 M sodium acetate and 800 μl of 100% (vol/vol) ethanol. RNA was collected by centrifugation at 14,000 × *g* for 15 min at 4°C. Pellets were air dried at room temperature and resuspended in 50 μl of TE buffer (10 mM Tris-HCl, 1 mM EDTA [pH 8.0]). RNA extracts were treated with TURBO DNase (Ambion, Thermo Fisher Scientific), and the absence of DNA was confirmed by quantitative PCR (qPCR) of the 16S rRNA gene using the Platinum SYBR Green qPCR SuperMix-UDG kit (Invitrogen, Thermo Fisher Scientific). RNA purity and concentration were evaluated on an ND-1000 spectrophotometer (Thermo Fisher Scientific). The extracted RNA was stored at –80°C before further analysis. Prior to mRNA enrichment, DNase-treated RNA for all soil replicas were pooled (equimolar basis) to generate a composite sample each for the control microcosms (one pooled C2 sample) and treatment microcosms (one pooled T2 sample). Composite RNA samples were applied to the subtractive hybridization method using the MicrobeExpress kit (Ambion, Thermo Fisher Scientific) to remove rRNA from the mRNA. Purified mRNA was eluted in 25 μl of TE buffer and reverse transcribed to cDNA using the SuperScript III first-strand synthesis system kit (Invitrogen, Thermo Fisher Scientific) following the manufacturer’s protocol. Community cDNA samples were subjected to library preparation using the Nextera Flex DNA library prep kit, followed by paired-end 150-bp sequencing on the Illumina HiSeq2500 platform at CD Genomics (New York). Sequencing yielded 41.2 million and 29.0 million paired reads for the treatment (T2) and control (C2) samples, respectively (Table S2).

### Sequence read processing and analysis.

Raw metagenomic and metatranscriptomic reads were assessed for quality and contamination using FastQC v0.11.7 (81) and MultiQC v1.0 (82) before and after processing. BBDuk v38.51 (https://sourceforge.net/projects/bbmap/) was used to trim Illumina adapter sequences, remove reads aligning to the PhiX genome, and trim low-quality bases (minimum quality score, 20), discarding reads less than 50 bp in length after trimming. Metatranscriptomic data were additionally filtered with BBDuk to remove reads corresponding to rRNA (Table S2).

### Community and diversity profiling.

Phylum-level community composition was determined for the metagenomes by GraftM v0.12.2 (83). 16S rRNA sequences were extracted and classified with the SILVA v132 (84) GraftM package, and taxonomic nomenclature was updated to reflect Genome Taxonomy Database (GTDB) v89 classifications (85). To calculate alpha diversity from the metagenomic data, operational taxonomic units (OTUs) were defined with SingleM v0.12.1 (https://github.com/wwood/singlem) by aligning to a database of single-copy ribosomal proteins. SingleM OTUs of the ribosomal protein gene *rplB* were clustered at 97% identity and used for downstream analysis. Shannon index (alpha diversity) and Chao1 (richness estimated) were calculated from the *rplB* OTU table in R v3.6.0 with the phyloseq v1.28.0 (86) estimate_richness function.

### Metabolic annotation of metagenomic short reads.

The metabolic capabilities of the desert soil communities were profiled by aligning metagenomic short reads to databases of metabolic markers. We used 37 manually curated protein databases as previously described (65) and an additional eight databases containing proteins from soil metagenomes annotated by hidden Markov models (HMMs), namely, terminal oxidases (CoxA, CydA, CyoA, and CcoN), formate dehydrogenase (FdhA), two NADH dehydrogenases (NuoF and NqrF), and ATP synthase (AtpA). For the metagenomes, forward reads at least 140 bp in length were aligned to the databases with the blastx function of DIAMOND v0.9.24 (87). DIAMOND alignment was performed with a query coverage threshold of 80% and a percent identity threshold of 80% (PsaA), 70% (PsbA), 75% (HbsT), 60% (CoxL, MmoX, AmoA, NxrA, RbcL, [FeFe]-hydrogenases, and group 4 [NiFe]-hydrogenases), or 50% (all other databases). To estimate the percentage of community members bearing each gene, gene abundance was calculated relative to the set of 14 universal single-copy ribosomal genes packaged with SingleM. Read counts were converted to reads per kilobase million (RPKM) to normalize to gene length and metagenome size. Reads aligning to the single-copy ribosomal genes (DIAMOND blastx, query coverage of 80% and bitscore threshold of 40) were converted to RPKM and averaged across the 14 genes to produce one number per sample representing the abundance of a single-copy gene carried by 100% of community members. Dividing the RPKM of each metabolic gene by this number gives the estimated percentage of the community with the gene, assuming one copy per genome.

### Metabolic annotation of metatranscriptomic short reads.

The relative abundance of metabolic marker gene transcripts in the metatranscriptomes was calculated by mapping the metatranscriptomic reads to a catalog of genes present in the metagenomes and quantifying expression in transcripts per million (TPM). Genes were predicted from the assembled contigs using Prodigal v2.6.3 (88) (-p meta). The nucleotide sequences of predicted genes were clustered with CD-HIT v4.8.1 (89) (cd-hit-est) at 99% to produce a nonredundant catalogue of genes present in the metagenomes. Salmon v1.1.0 (90) was used to quantify expression in the metatranscriptomic data using the gene catalogue as a “reference transcriptome” with default settings (salmon quant, –validateMappings). Genes in the gene catalogue were annotated with the 45 databases described above by aligning the protein sequences with DIAMOND BLASTP (thresholds as above), and TPM value outputs by Salmon were summed for predicted genes with the same annotation.

### Metagenomic binning and analysis.

To obtain sufficient sequences for metagenomic binning, four samples representing the desert soils in their native dry and wet states (C1, C3, T1, and T3) were subjected to additional deep metagenomic sequencing. They were sequenced on a 2 × 150-bp NextSeq500 run at the Australian Centre for Ecogenomics, yielding an average of 86.1 million paired reads per sample. For each of the four samples, raw reads sequenced from the two runs were pooled to produce one single cometagenome. Raw reads derived from the four cometagenomes and other four single-run metagenomes (C2, C4, T2, and T4) were quality controlled by clipping off primers and adapters and then filtering out artifacts and low-quality reads using the Read_QC module within the metaWRAP pipeline (91). The eight quality-controlled metagenomes were individually assembled using MEGAHIT v1.1.3 (92) (default parameters; C1, C3, T1, and T3) or SPAdes v3.13.0 4 (93) (metaSPAdes mode, default parameters; C2, C4, T2, and T4). Each of the eight assemblies was binned using the binning module (–metabat2 –maxbin2 –metabat1) and were consolidated using the Bin_refinement module (-c 50 -x 10) within the metaWRAP pipeline. Additionally, the eight metagenomes were coassembled using MEGAHIT v1.1.3 (–k-min 27 –kmin-1pass). The coassembly was binned using the binning module (–metabat2 –maxbin2) and consolidated using the Bin_refinement module (-c 50 -x 10) within the metaWRAP pipeline. The produced nine bin sets were aggregated and dereplicated using dRep v2.3.2 (94) (-comp 50 -con 10). After dereplication, a total of 39 medium-quality (95) metagenome-assembled genomes (MAGs) were obtained (Table S2). Each MAG was taxonomically assigned according to GTDB release 04-RS89 using GTDB-tk v0.3.3 (96). Genes within MAGs were predicted with Prodigal v2.6.3 (-p single) and annotated by alignment to the 45 databases described above with DIAMOND BLASTP and the previously described thresholds. Phylogenetic trees were constructed of complete and nearly complete amino acid sequences of the catalytic subunits of the group 1 and 2 [NiFe]-hydrogenases and ribulose 1,5-bisphosphate carboxylase/oxygenase (RuBisCO) retrieved from the binned and unbinned assembled reads. Trees were constructed in MEGA X (97) using the neighbor-joining method with evolutionary distances inferred by the Poisson distribution method, gaps treated with partial deletion, and bootstrapping with 200 replicates.

### Gas chromatography analysis.

H_2_ oxidation was measured through monitoring headspace H_2_ mixing ratios over time of sterilized 120-ml serum vials incubated with 4 g of desert soil. For the Australian desert samples, 4-g soil samples were collected from each microcosm at each sampling point (T1 to T4 and C1 to C4) and transferred into serum vials. For the Namib, Gobi, and Mojave desert samples, 4-g quantities of either native dry soils or experimentally wetted soils were transferred into the serum vials; soils were wetted by adding approximately 4 g of soil to an empty sterilized microcosm vessel with a mesh base, pouring 4 ml of Milli-Q water, and allowing covered vessels to drain at ambient temperature for 24 h. Serum vials were sealed with 0.1 M NaOH-treated butyl rubber stoppers, flushed with pressurized air, and injected with H_2_ (via 1% H_2_ in N_2_ gas cylinders; 99.999% pure) to achieve a final headspace mixing ratio of ∼10 ppmv. Headspace gas (2 ml) was sampled using a gas-tight syringe and measured as described previously (50) on a gas chromatograph containing a pulse discharge helium ionization detector (model TGA-6791-W-4U-2; Valco Instruments Company Inc.). Serum vials were incubated at either 28°C (Australian soils) or ambient temperatures (other soils). H_2_ oxidation was measured in both live samples and heat-killed controls (autoclaved at 121°C and 15 lb/in^2^ for 60 min). All sampling needles and syringes were flushed with helium (99.999% pure) prior to penetration of the vial septum to avoid interference from air and contamination from the previous sample. For long-term measurements, vials were supplemented with helium gas as necessary to allow headspace sampling to continue, with dilution factors accounted for when calculating H_2_ mixing ratios. First-order rate constants (*k*) for H_2_ oxidation were estimated using the exponential nonlinear regression function in GraphPad Prism 8.4.3.

### Carbon fixation assays.

Chemosynthetic and photosynthetic carbon fixation was measured for the Australian soil microcosm samples by tracing incorporation of ^14^C-labeled CO_2_. Gaseous ^14^CO_2_ (1% [vol/vol]) was generated by mixing 15 μl of radiolabeled sodium bicarbonate solution (NaH^14^CO_3_, 56.6 mCi nmol^−1^; Perkin Elmer) with equal volumes of 10% HCl solution in a sealed 5-ml glass vial and incubating it for 2 h at room temperature. Samples (0.5 g) of either live soil or heat-killed controls (autoclaved at 121°C and 15 lb/in^2^ for 60 min) were collected from each microcosm at each sampling point (T1 to T4 and C1 to C4). They were transferred to sterile 5-ml glass vials sealed with rubber septa. Each vial was injected with ^14^CO_2_ (1% [vol/vol]) gas to achieve a final initial headspace concentration of 400 ppmv, which otherwise comprised ambient air. The samples were incubated for 24 h at 28°C in technical quadruplicate under four conditions: (i) light plus ^14^CO_2_, (ii) light plus ^14^CO_2_ + 100 ppmv of H_2_, (iii) dark plus ^14^CO_2_, and (iv) dark plus ^14^CO_2_ plus 100 ppmv of H_2_. Samples incubated under dark conditions were covered with aluminum foil prior to incubation, while those incubated under light conditions were incubated under a light box. Unfixed ^14^CO_2_ was removed by transferring soils to 10-ml scintillation vials and suspending them in 2 ml of 10% acetic acid in ethanol. Samples were left to dry in a chemical fume hood at ambient temperature for 48 h. Approximately 10 ml of scintillation cocktail (EcoLume) was added and ^14^C counts were measured with a liquid scintillation counter (Tri-Carb 2810 TR; Perkin Elmer) operating at 93% efficiency. Background luminescence and chemiluminescence were corrected through internal calibration standards and by including an additional background vial. Background scintillation counts were subtracted from sample counts prior to calculations. The amount of ^14^CO_2_ fixed per sample was quantified as the mass of ^14^CO_2_ per mass of soil per time unit (picomoles per gram per day).

### Data availability.

All metagenomes, metatranscriptomes, and metagenome-assembled genomes are available at the Sequence Read Archive under BioProject accession number PRJNA647683.
