# Supplementary figures and images for: Hydrogen-Oxidizing Bacteria Are Abundant in Desert Soils and Strongly Stimulated by Hydration
Source: mSystems. 2020 Nov 17;5(6):e01131-20. doi: 10.1128/mSystems.01131-20 (PMC7677003; doi:10.1128/mSystems.01131-20)

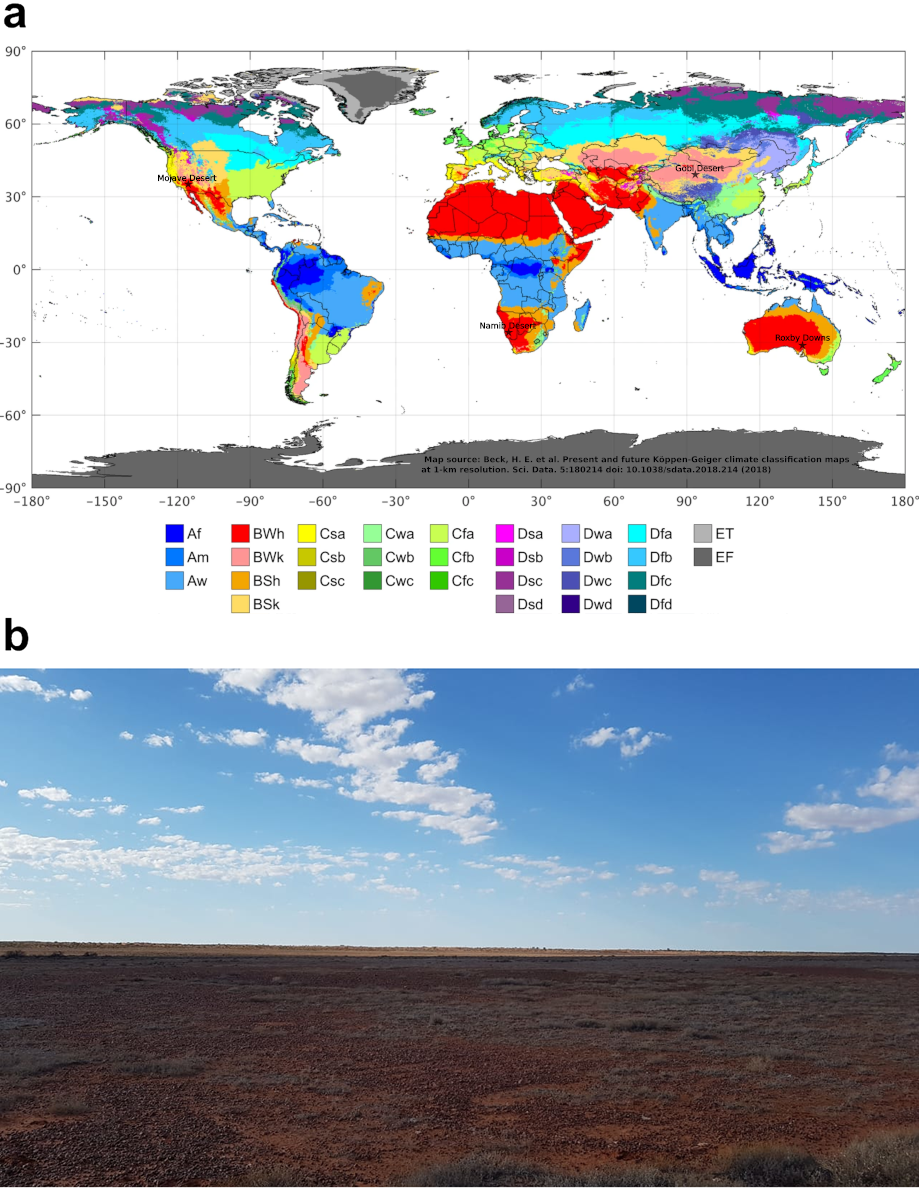

Supplement: FIG S1 [file mSystems.01131-20-sf001.tif]

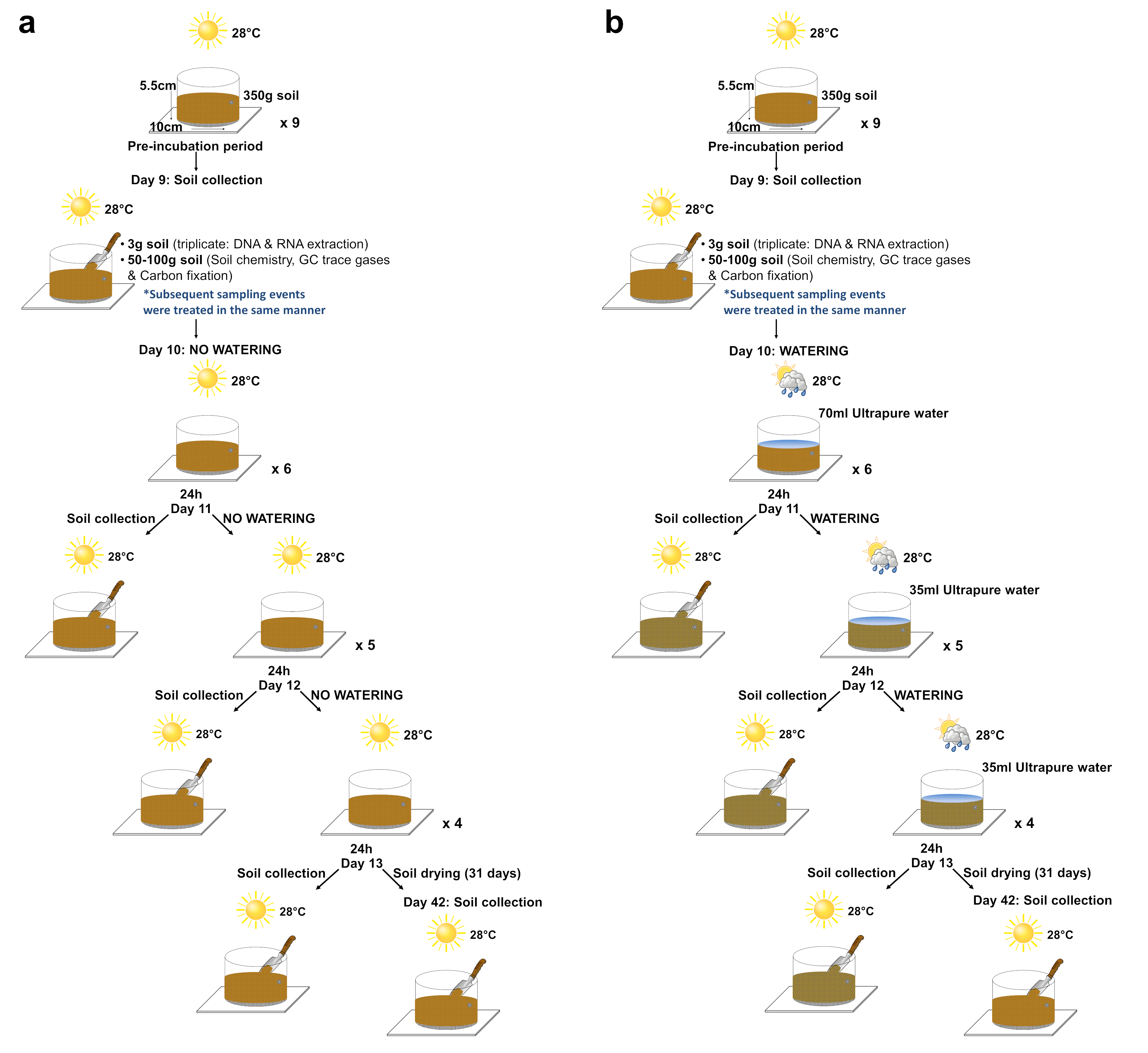

Supplement: FIG S2 [file mSystems.01131-20-sf002.tif]

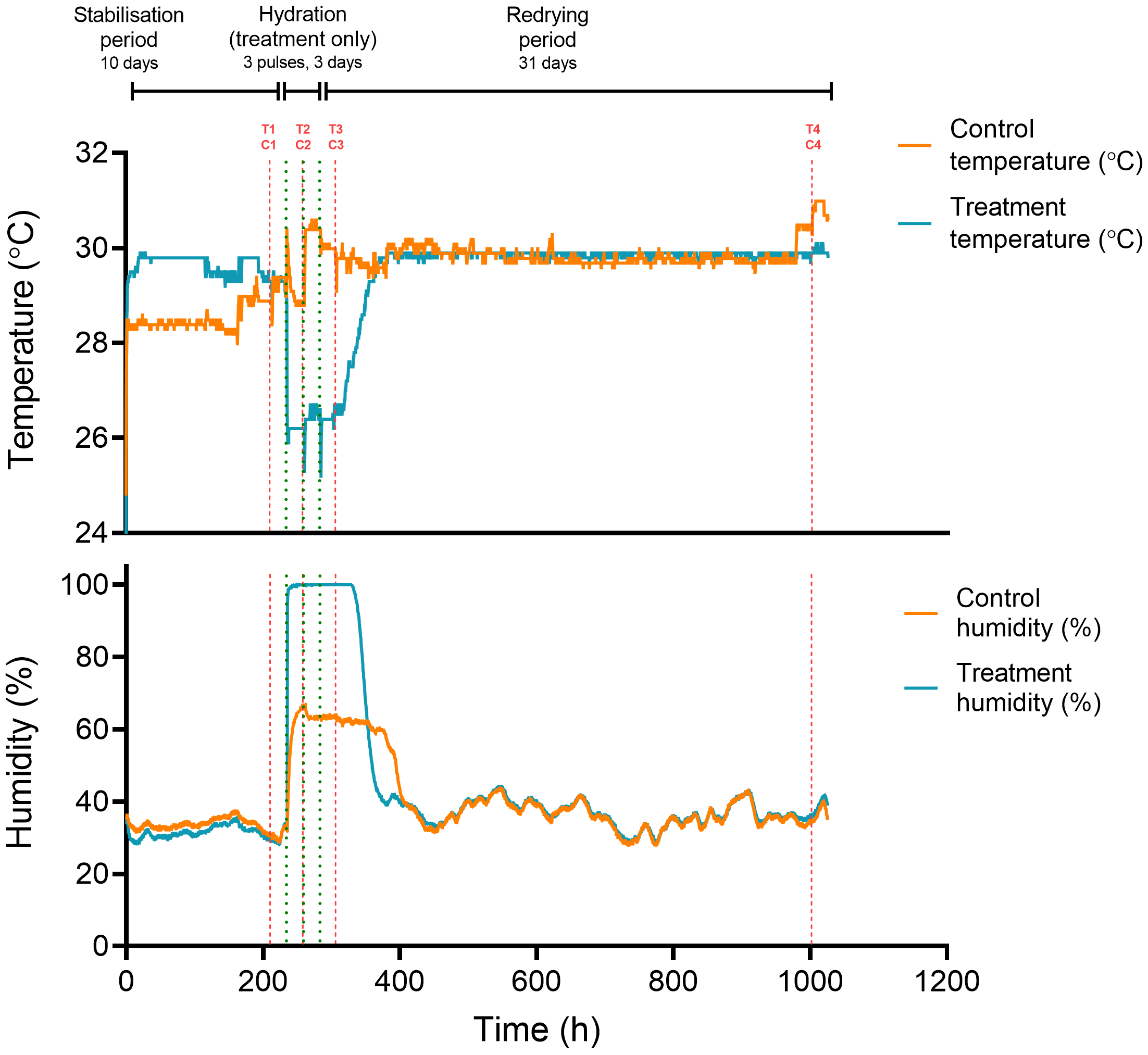

Supplement: FIG S3 [file mSystems.01131-20-sf003.tif]

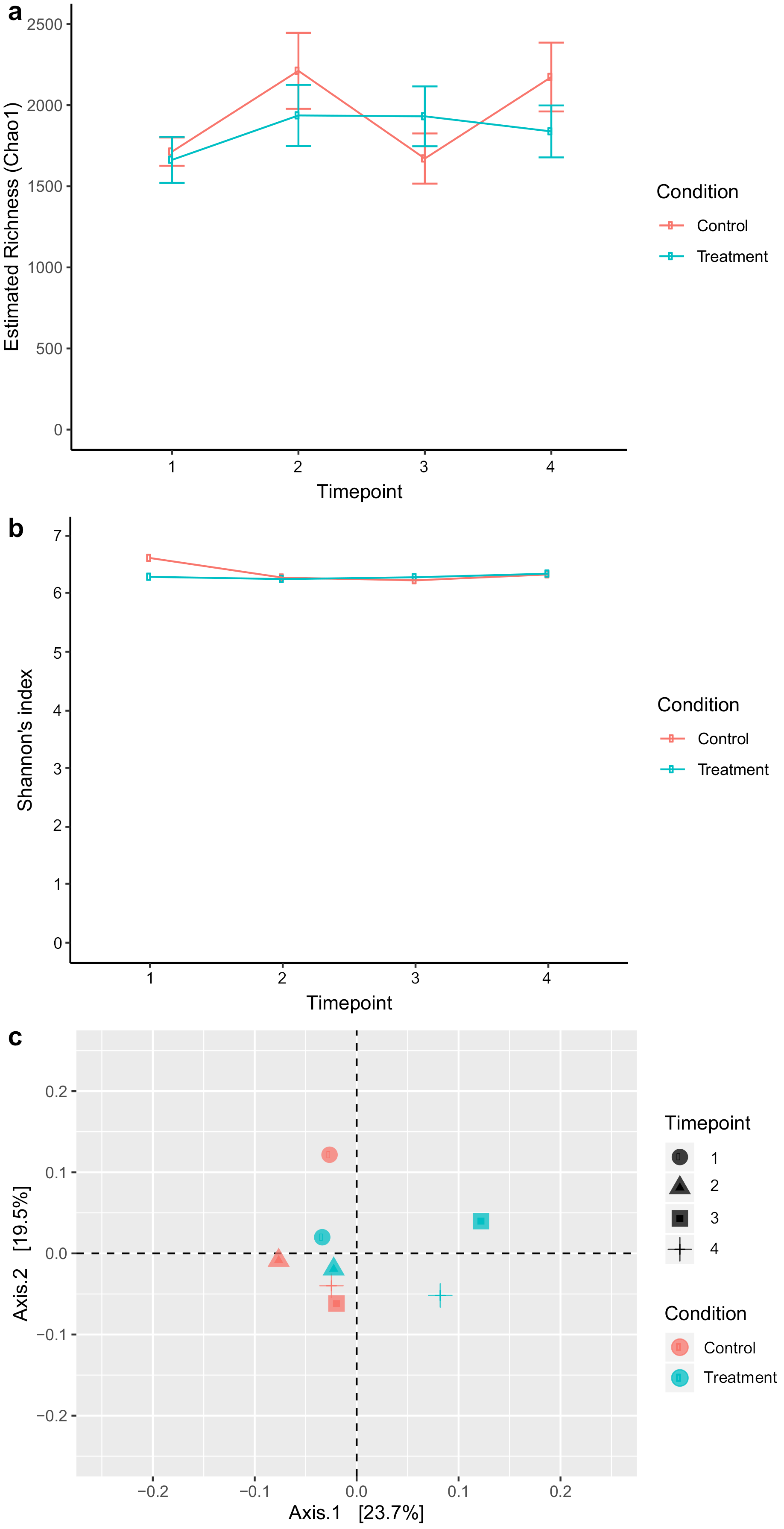

Supplement: FIG S4 [file mSystems.01131-20-sf004.tif]

Bootstrap values

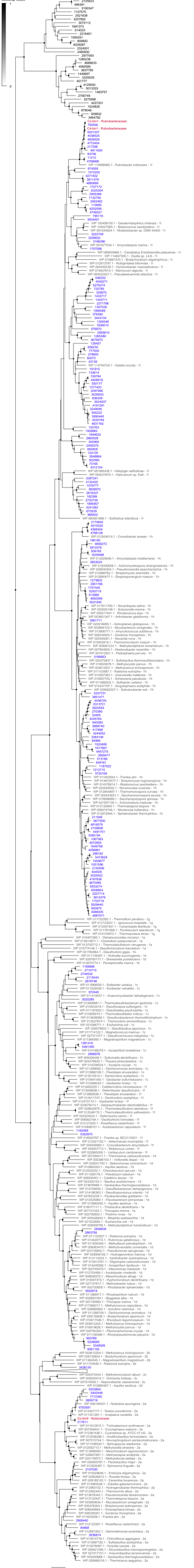

Supplement: FIG S5 [file mSystems.01131-20-sf005.pdf]

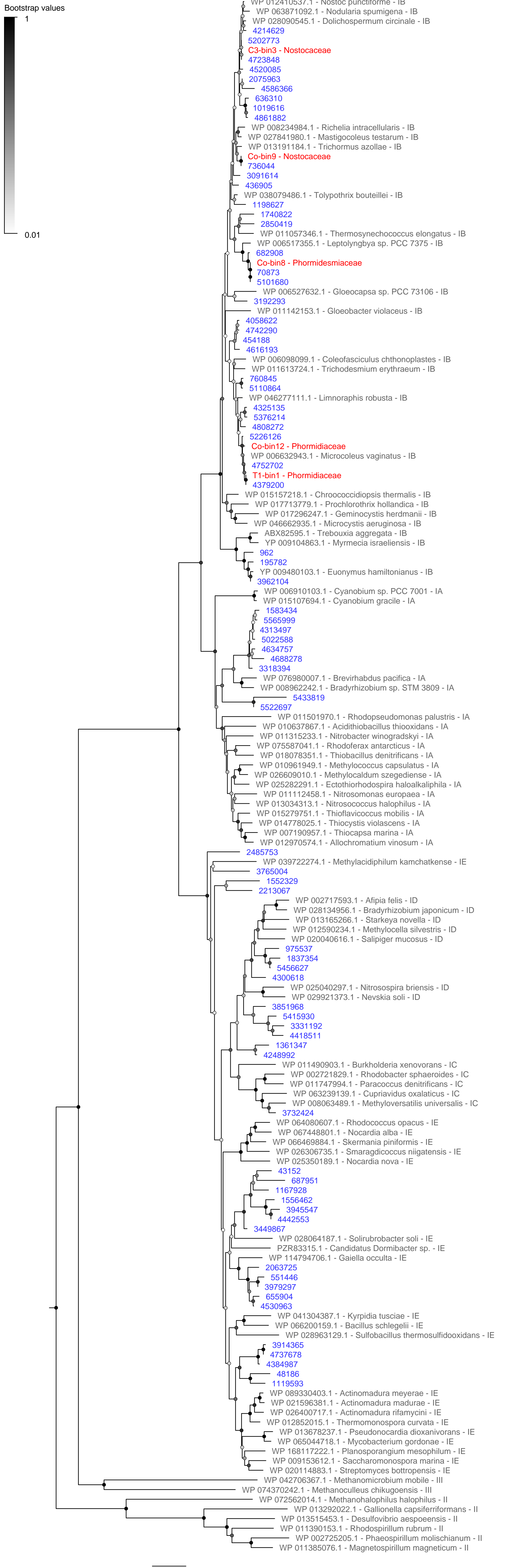

Supplement: FIG S6 [file mSystems.01131-20-sf006.pdf]
